# Global Deletion of the Prolactin Receptor Aggravates Streptozotocin-Induced Diabetes in Mice

**DOI:** 10.3389/fendo.2021.619696

**Published:** 2021-03-05

**Authors:** Gabriela Ramirez-Hernandez, Elva Adan-Castro, Nundehui Diaz-Lezama, Xarubet Ruiz-Herrera, Gonzalo Martinez de la Escalera, Yazmin Macotela, Carmen Clapp

**Affiliations:** Instituto de Neurobiología, Universidad Nacional Autónoma de México (UNAM), Querétaro, Mexico

**Keywords:** STZ-induced diabetes, prolactin, prolactin receptor null mice, beta-cell, glucose homeostasis

## Abstract

Prolactin (PRL) levels are reduced in the circulation of rats with diabetes or obesity, and lower circulating levels of PRL correlate with increased prevalence of diabetes and a higher risk of metabolic alterations in the clinic. Furthermore, PRL stimulates β-cell proliferation, survival, and insulin production and pregnant mice lacking PRL receptors in β-cells develop gestational diabetes. To investigate the protective effect of endogenous PRL against diabetes outside pregnancy, we compared the number of cases and severity of streptozotocin (STZ)-induced hyperglycemia between C57BL/6 mice null for the PRL receptor gene (*Prlr^-/-^*) and wild-type mice (*Prlr^+/+^*). STZ-treated diabetic *Prlr^-/-^* mice showed a higher number of cases and later recovery from hyperglycemia, exacerbated glucose levels, and glucose intolerance compared to the *Prlr^+/+^* mice counterparts. Consistent with the worsening of hyperglycemia, pancreatic islet density, β-cell number, proliferation, and survival, as well as circulating insulin levels were reduced, whereas α-cell number and pancreatic inflammation were increased in the absence of PRL signaling. Deletion of the PRL receptor did not alter the metabolic parameters in vehicle-treated animals. We conclude that PRL protects whole body glucose homeostasis by reducing β-cell loss and pancreatic inflammation in STZ-induced diabetes. Medications elevating PRL circulating levels may prove to be beneficial in diabetes.

## Introduction

Loss of pancreatic β-cells is a hallmark of diabetes type 1 and type 2, and factors that prevent β-cell loss or promote the generation of new β-cells are promising therapeutics (1). One such factor is the pituitary hormone prolactin (PRL). The PRL receptor (PRLR) is expressed in β-cells, where PRL stimulates proliferation, survival, insulin synthesis, glucose-induced insulin secretion, and glucose transporter 2 expression (2–6). The global *Prlr* null mouse (on a 129svJ background) has reduced islet size and density, decreased β-cell mass, and lowered production and secretion of insulin in response to glucose treatment (7). Also, mice lacking the PRLR in the pancreas (8) and specifically in β-cells, develop gestational diabetes (9).

The protective role of PRL in diabetes is further suggested by the facts that the circulating levels of PRL are reduced in rats with streptozotocin (STZ)-induced diabetes and diet-induced obesity (10–12), and low circulating PRL levels are deleterious for glucose homeostasis (13). Treatment of obese rats with PRL improves insulin sensitivity and challenging global *Prlr* null mice with an obesogenic diet exacerbates insulin resistance and glucose intolerance (12). Moreover, low serum PRL levels are associated with an increased prevalence of type 2 diabetes, insulin resistance, glucose intolerance, and fatty liver in the clinic (13–18). Nevertheless, the influence of the endogenous hormone on diabetes development and progression outside pregnancy remains unclear. PRL treatment reduces blood glucose levels in STZ-induced diabetes in mice (19) but high doses of the hormone induce β-cell apoptosis in 90% pancreatectomized rats (20). Here, we used the global *Prlr* null mice on a C57BL/6 background, a strain highly sensitive to the diabetogenic drug STZ (21, 22), and show that, when challenged with STZ, these animals are more susceptible to developing hyperglycemia and have a more severe hyperglycemic phenotype as revealed by higher blood glucose levels, glucose intolerance, islet loss, reduced β-cell number and survival, and increased pancreatic inflammation. These observations show the requirement of PRL signaling for glucose homeostasis and β-cell mass and function in STZ-induced diabetes and suggest the therapeutic benefit of PRL in diabetes.

## Materials and Methods

### Animals

Female C57BL/6 mice (5–7 weeks old), wild type (*Prlr^+/+^*) or null for the PRL receptor gene (*Prlr^-/-^*), were housed at 22°C under a 12/12 h light/dark cycle with free access to food and water. Females were selected because they are less susceptible to STZ than males (22, 23). All animal procedures and biosecurity measurements were approved by the Bioethics Committee of the Institute of Neurobiology of the National University of Mexico (UNAM) in accordance with the *Guide for the Care and Use of Laboratory Animals* published by the US National Institutes of Health (Eighth Edition, National Academy Press, Washington, D.C.). The use of streptozotocin followed the safety procedures established by the Office of Environmental Health and Safety of Virginia Commonwealth University (http://www.vcu.edu/oehs/chemical/biosafe/STZinfo.pdf).

### STZ-Induced Diabetes

Mice were injected with five IP doses of STZ (55 mg/kg in 10 mM citrate buffer, pH 4.5) (Sigma Aldrich, St. Louis, MO, USA) or vehicle (citrate buffer) after a 4-h fasting (24, 25) (one injection per day for five days). Animals with blood glucose levels >180 mg/dl one week after STZ treatment were considered diabetic (26, 27). Blood glucose levels (tail vein samples) and body weight (BW) were measured under 4-h fasting before STZ treatment and weekly during the following 11 weeks. Animals under 4-h fasting were euthanized by CO_2_ inhalation followed by decapitation at 2 or 11 weeks post-STZ. To avoid stress-induced PRL release, animals were handled daily during the seven days before sacrifice.

### Serum Measurements

Insulin and glucagon serum levels were quantified using commercial ELISA kits (Crystal Chem, Downers Grove, IL, USA).

### IP Glucose Tolerance Test

IP glucose tolerance test was performed 9 weeks after STZ treatment in control and diabetic *Prlr^+/+^* and *Prlr^-/-^* mice subjected to a 12-h fasting. Glucose levels were measured before and 15, 30, 60, 90, and 120 min after an IP injection of 2 g/kg of 50% (w/v) dextrose (PiSA Pharmaceuticals, Guadalajara, Mexico).

### Light Immunohistochemistry

Pancreatic tissues were collected 11 weeks post-STZ and divided into duodenal and splenic lobes. The splenic lobes of the pancreas were fixed in 10% formalin, and 5-µm-thick longitudinal sections were obtained. Three to four pancreatic sections per animal (each separated by 40 sections in between) were dehydrated and embedded in paraffin. Sections were deparaffinized at 60°C for 60 min and 99.5% xylene for 10 min, rehydrated with decreasing grades of ethanol (100, 95, 85, 70 and 50%) for 3 to 5 min, permeabilized (0.1% Triton X-100, 0.1% sodium citrate) for 30 min, and incubated with antigen-retrieval buffer (10 mM sodium citrate, 0.5% Tween 20, pH 6.0) in a pressure cooker for 3 min. Double immunohistochemistry for insulin and glucagon-labeled β-cells and α-cells, respectively, used a 1:500 dilution of rabbit polyclonal anti-insulin antibody (4590, Cell Signaling, Danvers, MA, USA) and mouse monoclonal anti-glucagon antibody (ab10988, Abcam, UK). The sections were then incubated with a 1:1000 dilution of biotinylated goat anti-rabbit IgG (Vector Laboratories, Burlingame, CA, USA) and peroxidase-coupled goat anti-mouse IgG (Jackson ImmunoResearch Laboratories, West Grove, PA, USA), and the reaction was developed using the Vectastain ABC-HRT Kit (Peroxidase) (PK-4001, Vector Laboratories, Burlingame, CA, USA) and DAB Substrate Kit (SK-4100, Vector Laboratories). Sections were counterstained with hematoxylin. The images were digitalized (Aperio ScanScope, Leica Biosystems, Buffalo Grove, IL, USA) using a 40X objective. The number of β- and α-cells was quantified from at least 15 islets per animal using the Image-Pro Plus software (version 7.0.9.591, Media Cybernetics, Inc, Bethesda, MD, USA) in a blinded manner.

### Fluorescence Immunohistochemistry

Paraffin-embedded sections (5-µm-thick) of the pancreatic splenic lobe were subjected to fluorescence immunohistochemistry as described previously (28) using a 1:200 dilution of mouse monoclonal anti-insulin antibody (I2018, Sigma Aldrich, St. Louis, MO, USA) or rabbit polyclonal antibodies against the proliferation marker, Ki67 (ab66155, Abcam, UK), and a 1:500 dilution of Alexa Flour 488 anti-mouse or Alexa Flour 555 anti-rabbit secondary antibodies (Invitrogen, Thermo Fisher Scientific, Waltham, MA, USA). Sections were cover-slipped with Vectashield mounting medium containing DAPI (Vector Laboratories, Inc., Burlingame, CA, USA) and observed under fluorescence microscopy (Microscope BX60F5, Olympus) using the 40X objective, and the fluorescence signal was quantified from at least 15 islets per animal using the Image-Pro Plus 5.1 software.

### Apoptosis Assay

**TUNEL**. Sections (5-μm-thick) from the splenic lobe of the pancreas were deparaffinized, rehydrated, permeabilized, and submitted to heat-induced antigen retrieval with citrate buffer (10 mM sodium citrate, 0.5% Tween 20, pH 6.0) in the microwave for 1 min. Apoptosis was detected by TUNEL using the in-situ cell death detection kit (Roche Diagnostics, Basel, Switzerland). Sections were then stained for insulin with a 1:200 dilution of the mouse monoclonal anti-insulin antibody described above and a 1:500 dilution of Alexa Flour 594 anti-mouse secondary antibody (Invitrogen, Thermo Fisher Scientific). The sections were mounted using Vectashield mounting medium containing DAPI (Vector Laboratories) and visualized under fluorescence microscopy (Microscope BX60F5, Olympus). **ELISA**. The duodenal lobe of the pancreas was fragmented, frozen in liquid nitrogen, and stored at −80°C. Fragments were homogenized in lysis buffer (5 mM Tris-HCl, 1 mM EGTA, 1mM EDTA, 1 mM sodium orthovanadate, 50 mM sodium fluoride, 100 mM sodium acid pyrophosphate, 250 mM sucrose, 1% Igepal Ca-630, pH 7.5) and total protein was determined by Bradford. Equal amounts of protein (60 μg) were evaluated by the cell death detection ELISA kit (Roche Diagnostics, Basel, Switzerland).

### RT-qPCR

Frozen pancreatic duodenal lobes were pulverized in liquid nitrogen, total RNA was extracted using TRIzol reagent (Invitrogen, Carlsbad, CA, USA), and cDNA was synthesized with the High-Capacity cDNA Reverse Transcription kit (Applied Biosystems, Foster City, CA, USA). PCR products were detected and quantified using Maxima SYBR Green qPCR Master Mix (Thermo Fisher Scientific) in a final reaction of 10 μl containing template and 0.5 μM of each of the primer pairs defined in **Supplementary Table 1**. The PCR data were analyzed by the 2^-ΔΔCT^ method and gene expression was normalized to the housekeeping gene cyclophilin A.

### Statistics

Statistical data analysis was performed using GraphPad Prism 6.0c software (Systat Software, San Jose, CA). When the distribution was normal and variances equal, the unpaired two-tailed Student’s *t*-test evaluated differences between two groups, whereas one-way ANOVA or two-way repeated measures ANOVA, as required, followed by Tukey’s *post-hoc* test compared means of multiple groups. The Log-Rank test compared the Kaplan-Meier survival analysis. Fisher’s test evaluated the association between numerical variables. The threshold for significance was set at P < 0.05.

## Results

### Deletion of the PRLR Increases the Cases, Reduces the Recovery, and Enhances the Severity of STZ-Induced Diabetes

**Figure 1:**
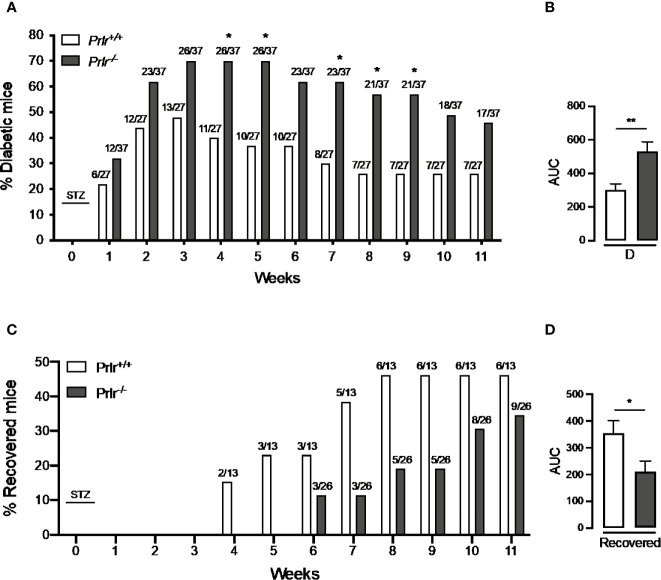
Absence of the prolactin receptor (PRLR) increases the number of cases and lowers their recovery from streptozotocin (STZ)-induced diabetes. **(A)** Number of diabetic wild-type mice (*Prlr^+/+^*) and null for the PRL receptor gene mice (*Prlr^-/-^*) relative to the total number of mice from each group injected with STZ. STZ was injected daily for five days in week 0. Diabetes is identified by blood glucose levels >180 mg/dl. Numbers above bars indicate the number of diabetic mice over total mice injected with STZ. **(B)** Area under the curve (AUC) was calculated from the percent of diabetic mice throughout the 11-week experimental period. **(C)** Number of animals recovered from diabetes (blood glucose < 180 mg/dl) relative to the total number of diabetic STZ-injected mice. **(D)** AUC was calculated from the number of cases recovered from diabetes throughout the 11-week experimental period. Statistical differences were examined using Fisher’s test to compare the proportion of cases with diabetes **(A)** or recovered from diabetes **(C)** each week; or by the unpaired two-tailed Student’s *t*-test **(B, D).** *P<0.05, **P<0.01.

**Figure 2:**
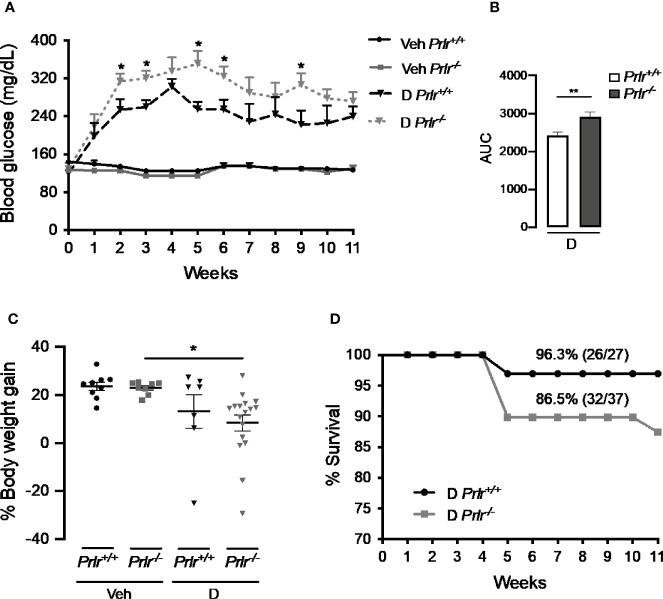
Absence of the prolactin receptor (PRLR) stimulates the severity of streptozotocin (STZ)-induced diabetes. **(A)** Blood glucose levels after 4-h fasting, at the onset of STZ or vehicle injection (week 0) and throughout the 11 weeks post-STZ or vehicle treatment in diabetic **(D)** or vehicle-injected (Veh) wild-type mice (*Prlr^+/+^*) and null for the PRL receptor gene mice (*Prlr^-/-^*). **(B)** Area under the curve (AUC) derived from the blood glucose levels averaged throughout the 11 weeks post-STZ. **(C)** Body weight gain after 11 weeks post-STZ injection (or vehicle) relative to body weight in week 0. Values are means ± SEM. The number of mice per group were: nine for Veh *Prlr^+/+^*, eight for Veh *Prlr^-/-^*, six to 13 for D *Prlr^+/+^*, and 12 to 17 for D *Prlr^-/-^*. **(D)** Kaplan-Meier survival analysis. Numbers above graphs indicate the percentage and numbers of surviving mice over total mice. Statistical differences were examined using one-way ANOVA followed by the Tukey *post hoc* test **(A, C)**, the unpaired two-tailed Student’s *t*-test **(B)**, or the Long-Rank test **(D)**. *P<0.05, **P<0.01.

To analyze whether the lack of PRL signaling influences the number of cases and the severity of STZ-induced diabetes, blood glucose was determined in *Prlr* ^-/-^ mice and *Prlr* ^+/+^ mice once a week for the 11 weeks after treatment with STZ. Animals were considered diabetic when blood glucose levels were above 180 mg/dl (26, 27). At week 3, the number of diabetic cases relative to the total number of STZ-injected mice was maximal in both *Prlr^+/+^* mice (48%) and *Prlr^-/-^* mice (70%), and dropped gradually thereafter, reaching a minimum at weeks 8 and 11 in *Prlr^+/+^* mice (26%) and *Prlr^-/-^* mice (46%), respectively (**Figure 1A**). The proportion of total diabetic cases, defined by the area under the curve (AUC), was higher (P=0.003) in *Prlr* null mice than in wild-type mice (**Figure 1B**). The number of animals recovered from diabetes (blood glucose <180 mg/dl) relative to the total number of diabetic STZ-injected mice is indicated in **Figure 1C**. Recovery started earlier (4 versus 6 weeks post-STZ) and was higher in *Prlr^+/+^* mice than in *Prlr^-/-^* mice. The proportion of total recovered cases, defined by the area under the curve (AUC), was lower (P=0.04) in *Prlr* null mice than in wild-type mice (**Figure 1D**). Once recovered, BW and blood glucose levels were similar between *Prlr^+/+^* and *Prlr^-/-^* mice (data not shown). Furthermore, diabetic STZ-injected *Prlr^-/-^* mice showed higher fasting glucose levels in the circulation, particularly during the first half of the experimental period (weeks 2 to 6 post-STZ) (**Figure 2A**). Glucose levels were similar between vehicle-injected groups. The AUC defined by glucose levels showed that glucose values were in average 21% higher in diabetic *Prlr^-/-^* mice than in diabetic *Prlr^+/+^* mice (299 ± 8.1 vs. 246 ± 12.7, P=0.002) (**Figure 2B**).

Vehicle-injected wild type mice and *Prlr* null mice gained around 4 g of BW from week 0 to week 11, which represented a 24% and a 23% increase in their BW, respectively (**Figure 2C**). The BW gain of diabetic STZ-injected *Prlr^+/+^* mice appeared lower (13%) than that of the vehicle-injected counterparts (24%), but the difference was not statistically significant. However, the reduction in BW gain observed between STZ-induced diabetic and vehicle-treated *Prlr^-/-^* mice (8% vs. 23%, respectively) was significant (P<0.04) (**Figure 2C**). Also, the survival rate throughout the 11 weeks of diabetes appeared lower in the absence of the PRLR (86% diabetic *Prlr^-/-^* mice and 96% diabetic *Prlr^+/+^* mice survived) (**Figure 2D**). These findings indicate that absence of PRL signaling increases the number of cases and worsens the progression and health outcome of STZ-induced diabetes in mice.

### Deletion of the PRLR Aggravates Glucose Metabolism in STZ-Induced Diabetes

**Figure 3:**
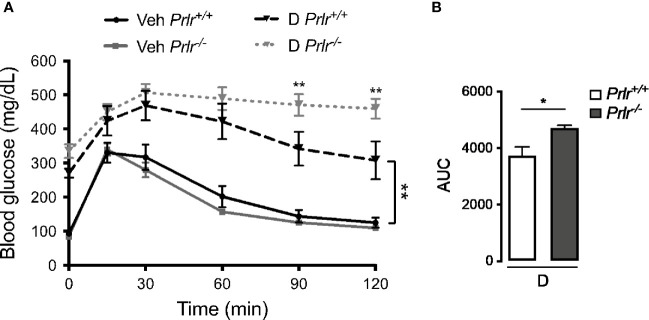
Glucose intolerance is exacerbated in the absence of the prolactin receptor (PRLR) in streptozotocin (STZ)-induced diabetes. **(A)** IP glucose tolerance test at 9 weeks post-STZ (or vehicle), in diabetic (D) or vehicle-treated (Veh) wild-type mice (*Prlr^+/+^*) and null for the PRL receptor gene mice (*Prlr^-/-^*). Time 0 shows blood glucose levels after 12-h fasting and immediately before glucose load. **(B)** Area under the curve (AUC) was calculated from the blood glucose levels of the IP glucose tolerance test throughout time. Values are mean ± SEM, n = 6 to 10 mice per group. Statistical differences were examined using two-way repeated measures ANOVA followed by the Tukey *post hoc* test **(A)**, or by the unpaired two-tailed Student’s *t*-test **(B)**. *P<0.05, **P<0.01.

Next, we performed the glucose tolerance test to evaluate the capacity of *Prlr^-/-^* mice to maintain glucose homeostasis. Vehicle-treated *Prlr^+/+^* mice and *Prlr^-/-^* mice showed a similar handling of a glucose load (**Figure 3A**). The glucose load resulted in higher blood glucose levels in the diabetic STZ-treated *Prlr^+/+^* mice that decreased more slowly than in the vehicle-treated mice. The glucose levels in the diabetic *Prlr^-/-^* mice were similar to those of the diabetic *Prlr^+/+^* mice at the peak but did not decline afterwards during the assay (**Figure 3A**). Total glucose levels, defined by the AUC, were higher in diabetic mice lacking the PRLR (**Figure 3B**). This indicated that diabetic mice null for the PRLR exhibit worse glucose intolerance than diabetic wild type mice.

### Deletion of the PRLR Reduces Islet and β-Cell Densities and Increases α-Cell Density in STZ-Induced Diabetes

**Figure 4:**
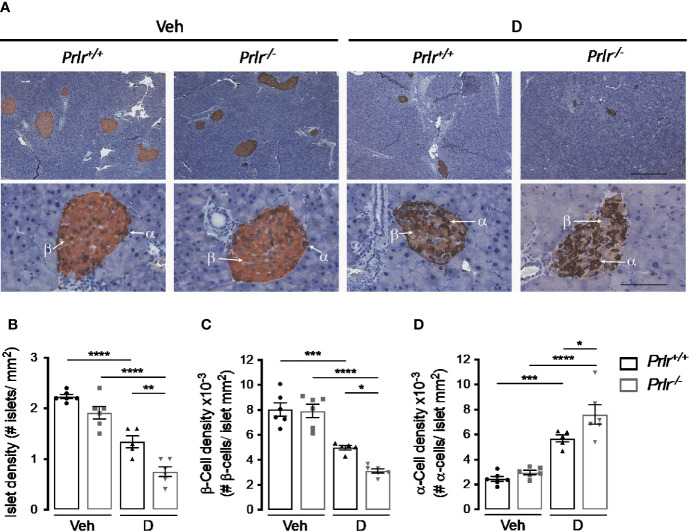
Absence of the prolactin receptor (PRLR) reduces islet and β-cell densities and increases α-cell density in streptozotocin (STZ)-induced diabetes. **(A)** Representative sections of pancreatic tissue from diabetic **(D)** and vehicle-treated (Veh) wild-type mice (*Prlr^+/+^*) and null for the PRL receptor gene mice (*Prlr^-/-^*) at 11 weeks post-STZ or vehicle. β-Cells (light brown) and α-cells (dark brown) were immunostained for insulin and glucagon, respectively, and counterstained with hematoxylin. Scale bar is 200 µm and 50 µm in upper and bottom panels, respectively. **(B)** Number of islets, **(C)** β-cells, and **(D)** α-cells per mm^2^ of pancreatic tissue. Values are means ± SEM, n = 5 to 6 mice per group. Statistical differences were examined by one-way ANOVA followed by the Tukey *post hoc* test **(B–D)**. *P<0.05, **P < 0.01, ***P < 0.001, ****P < 0.0001.

To investigate the basis for the worsening of diabetes in *Prlr^-/-^* mice, we examined the histology of the endocrine pancreas 11 weeks after inducing diabetes with STZ. Islets immunostained with anti-insulin and anti-glucagon antibodies showed that their number per area of the pancreas (islet density) was similar between the vehicle-treated groups and reduced (P<0.0001) in STZ-induced diabetes (**Figures 4A, B**). The diabetes-induced reduction in islet density was more severe (P=0.0031) in *Prlr^-/-^* mice than in *Prlr^+/+^* mice (**Figures 4A, B**).

Additional morphometric analysis evaluated the localization and content of insulin-positive β-cells and glucagon-positive α-cells within the islets (**Figure 4A**). The findings confirmed the distribution of β-cells and α-cells in the core and periphery of rodent islets, respectively (29) (**Figure 4A**). As expected (30), the diabetic condition was associated with a reduction in the proportion of β-cells and with more α-cells that were distributed in the core of the islet compared to vehicle-injected controls (**Figures 4A, C, D**). However, the changes in density and location of β-cells and α-cells in the diabetic condition were intensified in the absence of the PRLR (**Figures 4A, C, D**). These observations suggested that lack of PRL signaling increases the loss of β-cells and the gain of α-cells which, in turn, worsen STZ-induced diabetes.

### Deletion of the PRLR Reduces Serum Insulin Levels in STZ-Induced Diabetes

**Figure 5:**
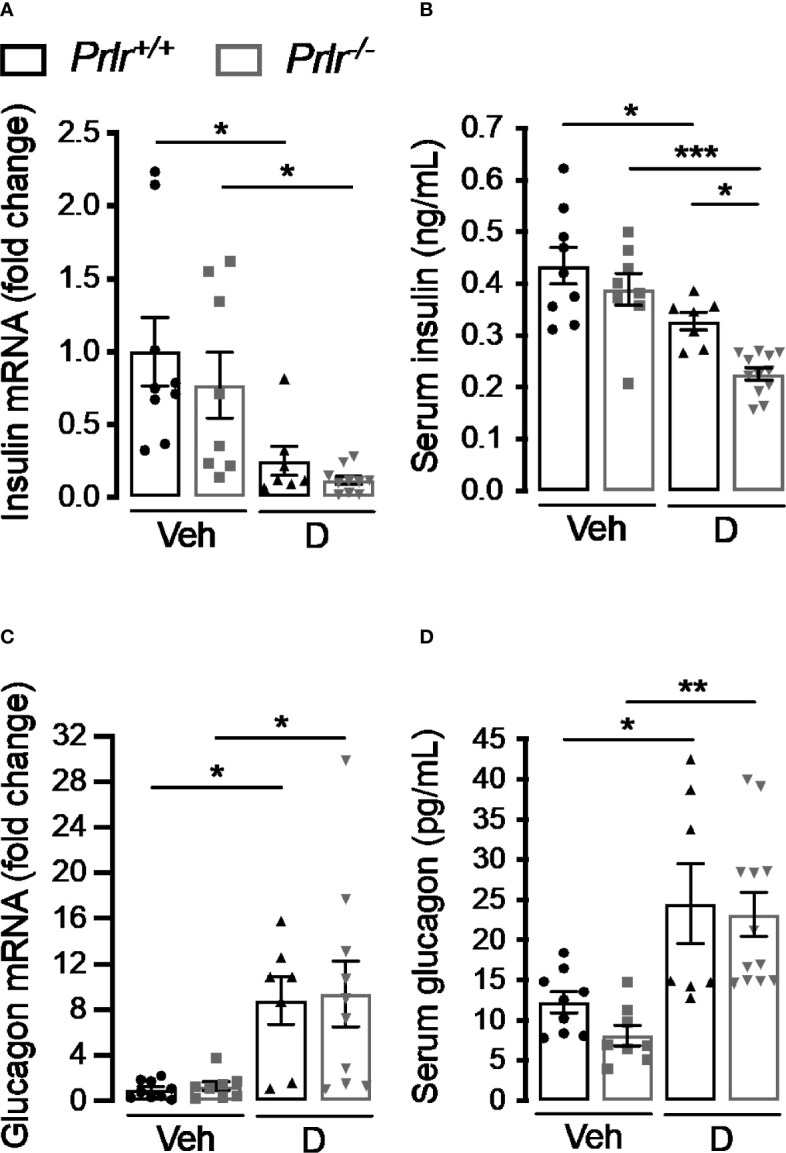
Absence of the prolactin receptor (PRLR) reduces serum insulin levels in streptozotocin (STZ)-induced diabetes. **(A, C)** Pancreatic mRNA expression and **(B, D)** serum levels of insulin and glucagon in 4 h-fasted diabetic **(D)** and vehicle-treated (Veh) wild-type mice (*Prlr^+/+^*) and null for the PRL receptor gene mice (*Prlr^-/-^*) at 11 weeks post-STZ or vehicle. Values are means ± SEM, n = 7 to 12 mice per group. Statistical differences were examined by one-way ANOVA followed by the Tukey *post hoc* test **(A–D)**. *P<0.05, **P < 0.01, ***P < 0.001.

To further investigate the functional implication of the histological changes in the endocrine pancreas of diabetic *Prlr^-/-^* mice, we determined the pancreatic expression and circulating levels of insulin and glucagon 11 weeks after inducing diabetes with STZ. Consistent with the loss of β-cells, insulin mRNA and circulating levels decreased in the diabetic groups compared with the vehicle-injected counterparts (**Figure 5A**). Insulin expression appeared lower in diabetic *Prlr^-/-^* mice than in diabetic *Prlr^+/+^* mice, although the difference was not statistically significant. However, the diabetes-induced reduction in circulating insulin levels was significantly higher in mice null for the PRLR (**Figure 5B**).

The increase in α-cell density found in the diabetic condition was mirrored by enhanced pancreatic glucagon mRNA expression and circulating levels in diabetic animals compared with the vehicle-treated groups (**Figures 5C, D**). The increases were of similar magnitude in both genotypes, in spite of *Prlr^-/-^* mice having higher numbers of α-cells per islet area (**Figures 4A, D**). This apparent contradiction may be explained by the lower islet density that occurs in *Prlr^-/-^* mice which could have counteracted the increase in glucagon production and release due to the higher number of α-cells per islet.

### Deletion of the PRLR Reduces β-Cell Proliferation in STZ-Induced Diabetes

**Figure 6:**
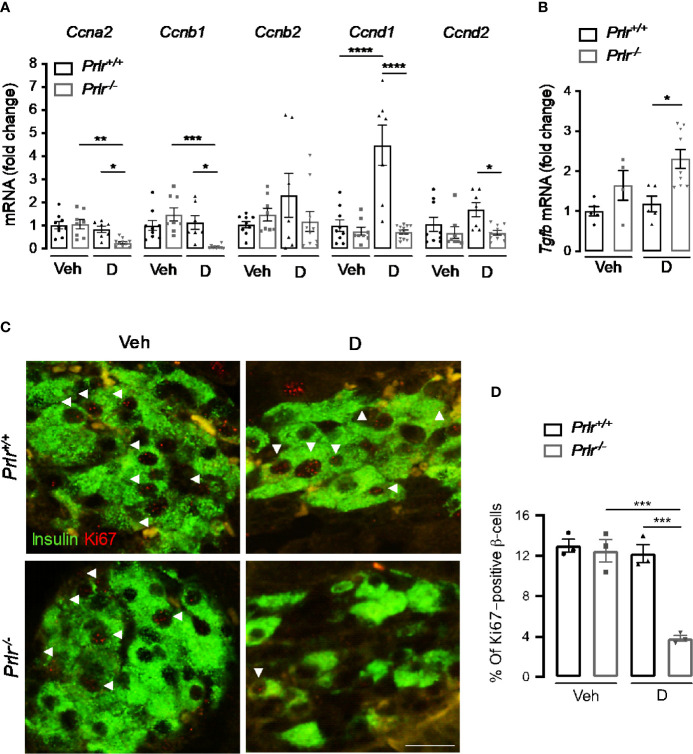
Absence of the prolactin receptor (PRLR) decreases β-cell proliferation in streptozotocin (STZ)-induced diabetes. **(A)** Messenger RNA expression of cyclin (*Cnna2*, *Ccnb1*, *Ccnb2*, *Ccnd1*, *Ccnd2)* and **(B)** transforming growth factor β (*Tgfb)* genes in the pancreas of diabetic (D) and vehicle-treated (Veh) wild-type mice (*Prlr^+/+^*) and null for the PRL receptor gene mice (*Prlr^-/-^*) at 11 weeks post-STZ or vehicle. **(C)** Representative fluorescence images of pancreatic sections immunostained for insulin (green cytoplasmic stain) and Ki67 (red nuclear stain), scale bar = 20 µm. **(D)** Percentage of cells doubled stained for Ki-67 and insulin relative to the total number of insulin-positive cells. Values are means ± SEM, n = 3 to 12 mice per group. Statistical differences were examined by one-way ANOVA followed by the Tukey *post hoc* test **(A, B, D)**. *P<0.05. **P < 0.01, ***P < 0.001, ****P < 0.0001.

STZ causes a partial destruction of β-cells (31, 32), and the proliferation of residual β-cells promotes disease recovery (33). Because reversal of diabetes slows down in diabetic *Prlr^-/-^* mice (**Figure 1**), we investigated whether β-cell proliferation was reduced in the absence of PRL signaling 11 weeks after STZ-induced diabetes. First, we measured the mRNA expression of genes *Ccna2, Ccnb1, Ccnb2, Ccnd1*, and *Ccnd2* (respectively encoding for cyclins A2, B1, B2, D1, and D2) implicated in β-cell proliferation (34). Pancreatic mRNA expression of *Ccna2, Ccnb1, Ccnd1, and Ccnd2* were downregulated in the diabetic *Prlr^-/-^* mice compared to diabetic *Prlr^+/+^* mice (**Figure 6A**). Moreover, the mRNA expression of the gene (*Tgfb*) encoding transforming growth factor β, an inhibitor of β-cell proliferation and differentiation (35), was higher in the pancreas of diabetic *Prlr^-/-^* mice relative to diabetic *Prlr^+/+^* mice (**Figure 6B**). Gene expression was similar between vehicle-treated groups. These findings suggested that β-cell proliferation decreases in PRLR-null mice. We confirmed this possibility by evaluating β-cell proliferation with double-label immunofluorescence using antibodies against the proliferation marker Ki-67 and anti-insulin antibodies. Consistent with the low turnover of β-cells in the adult (36), few Ki-67 proliferating β-cells were detected in all conditions (**Figure 6C**). The quantitation of Ki-67- and insulin-positive cells relative to all insulin-positive cells showed that β-cell proliferation was 68.7% lower in diabetic *Prlr* null mice than in their wild type diabetic counterparts (P=0.003) (**Figure 6D**). We conclude that reduced β-cell proliferation contributed to the β-cell loss that leads to a slower recovery from STZ-induced diabetes in *Prlr* null mice.

### Deletion of the PRLR Increases Apoptosis of β-Cells and Pancreatic Inflammation Soon After STZ Treatment

**Figure 7:**
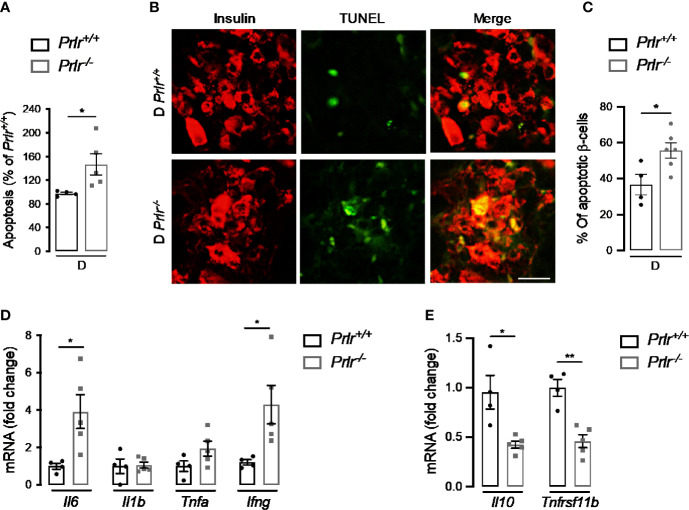
Absence of prolactin receptor (PRLR) increases β-cell apoptosis and pancreatic inflammation at early streptozotocin (STZ)-induced diabetes. **(A)** Apoptosis evaluated by ELISA in the pancreas of diabetic wild-type mice (*Prlr^+/+^*) and null for the PRL receptor gene mice (*Prlr^-/-^*) at 2 weeks post-STZ. The number of animals is indicated inside bars. **(B)** Representative fluorescence images of pancreatic sections double stained for apoptosis (TUNEL, green) and β-cells (insulin, red). Scale bar = 20 µm. **(C)** Percentage of cells doubled stained for insulin and TUNEL relative to the total number of insulin-positive cells. The number of animals indicated inside bars. **(D)** Messenger RNA expression of genes encoding pro-inflammatory cytokines (*Il6, Il1b, Tnfa, Infg*), and **(E)** anti-inflammatory cytokine (*Il10*), and the β-cell mitogen, osteoprotegerin (*Tnfrsf11b).* Four to five mice per group. Values are means ± SEM. Statistically significant differences were determined by the unpaired two-tailed Student’s *t*-test **(A, C–E)**. *P<0.05, **P < 0.01.

Finally, we aimed to evaluate whether the higher number of diabetic cases in *Prlr^-/-^* mice (**Figure 1**) involved an increase in β-cell apoptosis and inflammation due to the early cytotoxic effect of STZ (37). To this end, we evaluated apoptosis in the pancreas of mice 2 weeks after inducing diabetes with STZ. Apoptosis, determined by DNA fragmentation measured by ELISA, was 45% higher in diabetic *Prlr* null mice than in their wild type diabetic counterparts (P=0.04) (**Figure 7A**). Enhanced apoptosis of β-cells was confirmed by the increase in cells colocalizing TUNEL and insulin staining in diabetic *Prlr^-/-^* mice relative to diabetic *Prlr^+/+^* mice (**Figures 7B, C**). Therefore, loss of the PRLR enhanced the early apoptosis of β-cells in response to STZ that leads to the diabetogenic effect of the drug.

STZ elicits the apoptosis of β-cells through cytotoxic free radical generation (38), but also by promoting cytokine-mediated inflammatory responses in the pancreas (37, 39, 40). The pancreatic mRNA expression of pro-inflammatory cytokines (interleukin-6 and interferon-*γ*) was upregulated (**Figure 7D**), and that of an anti-inflammatory cytokine (interleukin-10) and the β-cell mitogen osteoprotegerin (encoded by the tumor necrosis factor receptor superfamily member 11B gene or *Tnfrsf11b*) downregulated (**Figure 7E**) in diabetic *Prlr^-/-^* mice compared with diabetic *Prlr^+/+^* mice. These findings suggested that loss of PRLR increases local inflammation in the pancreas, thereby contributing to β-cell loss and islet dysfunction in STZ-induced diabetes.

## Discussion

Lactogens (PRL and placental lactogen) are elevated in pregnancy, and studies have shown that these hormones contribute to the increase in insulin secretion and β-cell growth needed to accommodate the metabolic demand placed on the mother. Lactogens signal through the PRLR to stimulate pancreatic β-cell proliferation and insulin levels *in vitro* (41) and *in vivo* (42), and the specific deletion of the PRLR in pancreatic tissue (8) and in β-cells leads to failure to expand β-cell mass during pregnancy and to gestational diabetes (9). Yet it is unclear whether the action of endogenous PRL on glucose homeostasis protects against diabetes outside pregnancy. Two preclinical studies have addressed the effect of PRL administration on diabetes outside pregnancy (20, 43). One of them was carried out 21 years ago and showed that PRL administration reduced hyperglycemia in male mice treated with STZ but that the use of bromocriptine to reduce endogenous PRL levels had no effect (19). The study did not measure PRL levels nor provided support for a physiological action beyond a pharmacological effect. More recently, treatment with high and low doses of PRL were shown to have no effect and to improve insulin secretory activity in 90% pancreatectomized diabetic male rats, respectively, whereas the high but not the low PRL dose induced β-cell apoptosis in this model (20). These findings indicated that the dose of PRL can have opposite effects on glucose homeostasis and generated uncertainty regarding the role of endogenous PRL. In contrast to studying the effect of exogenous PRL, here we have evaluated the action of the endogenous hormone by using *Prlr* null mice. We found that lack of PRLR signaling increases the prevalence and severity of STZ-induced diabetes by enhancing β-cell dysfunction (reduced proliferation, survival, and insulin production). To the best of our knowledge, our report shows for the first time that endogenous PRL ameliorates diabetes beyond pregnancy.

Type 1 and type 2 diabetes occur when β-cells are unable to meet the increased demand for insulin due to insulin deficiency and insulin resistance, respectively. The STZ-induced diabetes model in rodents allows for the study of potential interventions based on using β-cell growth factors to enhance functional β-cell mass and insulin secretion. STZ is toxic to β-cells, as it is taken up *via* the cell membrane glucose transporter 2 and causes DNA and protein alkylation leading to β-cell apoptosis, hyperglycemia, and inflammation (32, 37, 44). Because PRL stimulates β-cell mass and function, we sought to evaluate whether STZ-induced diabetes is aggravated in mice null for the PRLR.

We used *Prlr* null mice on the C57BL/6 genetic background, a strain suitable for studies of glucose homeostasis (45) that is highly sensitive to STZ (21, 22). These mice showed no alterations in glucose metabolism under control conditions as revealed by normal glucose tolerance, islet and β-cell densities, pancreatic insulin expression, and circulating insulin levels. These findings are similar to those reported in the global heterozygous- (46) or β-cell-specific (9) *Prlr* null mice on a C57BL/6 background, but contrast with those on a 129/SvJ background, where global deletion of the PRLR associated with glucose intolerance, reduced islet density and β-cell mass, and lower pancreatic insulin expression and release (7), and also in mice on a mixed C57BL/6-129/SvJ background with deletion of the PRLR in the pancreas that show reduced islet number and β-cell mass, but normal glucose levels and tolerance (8). These contrasting findings are consistent with reported differences in glucose metabolism between both mouse strains (45) and indicate that the genetic background influences the metabolic outcome of PRL action. However, in spite of having no metabolic alterations under normal conditions, when challenged by pregnancy (9, 46) or STZ-induced diabetes (present findings), C57BL/6 *Prlr* null mice were unable to maintain whole body glucose homeostasis and β-cell mass and function, indicating that PRL signaling plays a fundamental role in glucose homeostasis regardless of the genetics.

In type 1 diabetes, β-cell death due to autoimmune destruction occurs at the early stages of the disease and leads to a rapid reduction in insulin levels and reciprocal hyperglycemia (47). Interestingly, a spontaneous partial recovery phase, in which β-cell function and insulin levels increase, occurs in some patients (48). The STZ mouse model mimics some of these basic aspects of the disease in humans. Islet apoptosis starts early, 2 days post STZ, and peaks 2 to 3 weeks later in association with maximal hyperglycemia (33, 37); also, some animals are able to return to normoglycemic levels (33, 49). Our study confirmed these dynamics and showed that both the number of cases and the recovery from STZ-induced diabetes are worsened in the absence of the PRLR. Furthermore, lack of PRL signaling was associated with enhanced β-cell apoptosis, pancreatic upregulation of the pro-inflammatory cytokines IL-6 and INF*γ*, and downregulation of the anti-inflammatory cytokine IL-10 shortly after STZ treatment. Because, STZ elicits the apoptosis of β-cells through the generation of cytotoxic free radicals (38) and the promotion of pancreatic inflammatory responses (37), our findings suggest that PRL counteracts diabetes by blocking oxidative stress and pancreatic inflammation early after STZ treatment. Supporting this notion, PRL has anti-oxidant (50) and anti-inflammatory (51) properties in different tissues including β-cells, where it protects against nitric oxide donor-, H_2_O_2_-, and cytokine-induced apoptosis *in vitro* (52).

Consistent with the reduced survival of β-cells, diabetes was exacerbated 11 weeks after STZ treatment in the absence of PRL signaling. *Prlr* null mice showed higher glucose circulating levels, reduced BW gain, decreased animal survival, and increased glucose intolerance. These observations complement the findings of previous studies showing that PRL treatment (19) and long-term pancreatic-induced expression of mouse placental lactogen-1 (42) counteract STZ-induced hyperglycemia, but where the underlying physiology resulting in lowered glucose levels was not defined. Exacerbation of STZ-induced diabetes in *Prlr* null mice was associated with reduced islet density, number of β-cells, insulin expression, and circulating insulin levels. The loss of β-cells not only reflected reduced survival but also lowered the ability to proliferate. Diabetic *Prlr* null mice showed less Ki67-positive proliferating β-cells and impaired pancreatic expression of the genes encoding for G1/S cyclins (*Ccna2, Ccnd1* and *Ccnd2*) and G2/M cyclins (*Ccnb1*) that are downregulated in the β-cell-specific *Prlr* null mice (9) or upregulated in response to lactogens (34).

The mechanism by which PRL stimulates β-cell replication involves the expression of osteoprotegerin (OPG), a bone-related decoy receptor that acts as a β-cell mitogen by binding and neutralizing the action of receptor activator of nuclear factor KB ligand (RANKL), a brake in β-cell proliferation (53). In support of this mechanism, we found that OPG is downregulated in the pancreas of diabetic *Prlr* null mice, and have reported that deletion of the PRLR promotes the upregulation of the RANKL/RANK/OPG system that leads to bone loss in inflammatory arthritis (54). In addition, TGFβ may be a contributing mechanism since its pancreatic expression increased in diabetic *Prlr* null mice, and the upregulation and downregulation of TGFβ/SMAD signaling inhibits and stimulates β-cell replication, respectively (35, 55). Whether PRL-mediated inhibition of TGFβ is a direct or indirect mechanism warrants further investigation.

Our work confirmed the expansion and central distribution of α−cells within the islet of STZ-induced diabetic mice (30) and showed that these changes are exacerbated in *Prlr* null mice. However, increased α−cell density in the absence of PRL signaling was not associated with higher pancreatic expression and circulating levels of glucagon. This apparent contradiction may relate to fact that the increase in α−cells per islet is counteracted by the reduced number of pancreatic islets in *Prlr* null mice. Expansion of α−cells in the diabetic state may result from the dedifferentiation of intra-islet β-cells to α−cells and a regeneration of α−cells (56). The expression and effect of the PRLR on alpha cells have not been described and further research is needed to elucidate whether α−cells are direct targets of PRL in diabetes.

The finding that the PRL receptor ameliorates diabetes severity is in line with the role of this hormone as a guardian of metabolic homeostasis (13). It was recently recognized that the beneficial outcome of PRL on metabolism depends on its circulating levels being kept within a range (between 7 and 100 ng/mL) defined as “homeostatic functionally increased transient prolactinemia” or HomeoFIT-PRL levels (13). This definition is based on multiple experimental and clinical studies showing that PRL values that are not only below (13–17) but also above this level are associated with metabolic disease. Indeed, circulating PRL levels higher than 100 ng/mL found in patients with prolactinomas or after treatment with hyperprolactinemia-inducing drugs, and in animal hyperprolactinemic models, result in metabolic alterations such as obesity, insulin resistance, non-alcoholic fatty liver disease, and glucose intolerance (57–60). Based on these observations and those of our current findings, we conclude that drugs elevating PRL levels within the HomeoFIT-PRL range may prove to be a promising therapy for diabetes.

## Data Availability Statement

The raw data supporting the conclusions of this article will be made available by the authors, without undue reservation.

## Ethics Statement

The animal study was reviewed and approved by Bioethics Committee of the Institute of Neurobiology of the National University of Mexico (UNAM).

## Author Contributions

GR-H, YM, and CC designed the research. GR-H, EA-C, ND-L, and, XR-H performed the research. GR-H, YM, and CC analyzed the data. GM discussed data, provided scientific expertise and contributed to writing the manuscript. YM and CC directed the study and wrote the manuscript. All authors contributed to the article and approved the submitted version.

## Funding

This study was supported by grants 247164 and 289568 from the Consejo Nacional de Ciencia y Tecnología (CONACYT) and by the Universidad Nacional Autónoma de México (UNAM) grant 405PC to CC. GR-H is a doctoral student from Programa de Doctorado en Ciencias Biomédicas, Universidad Nacional Autónoma de México (UNAM) and received CONACYT fellowship 589293.

## Conflict of Interest

The authors declare that the research was conducted in the absence of any commercial or financial relationships that could be construed as a potential conflict of interest.

## Abbreviations

BW, body weight; PRL, prolactin; PRLR, Prolactin receptor; *Prlr^-/-^*, prolactin receptor null mice; *Prlr^+/+^*, wild type mice (same genetic background as *Prlr^-/-^* mice); STZ, streptozotocin.
