# Supplementary material for: Global Deletion of the Prolactin Receptor Aggravates Streptozotocin-Induced Diabetes in Mice
Source: Front Endocrinol (Lausanne). 2021 Mar 5;12:619696. doi: 10.3389/fendo.2021.619696 (PMC7973366; doi:10.3389/fendo.2021.619696)
Supplement: Supplementary file 1 [file Table_1.docx]

Supplementary Table 1. Oligonucleotide sequences used for RT-PCR

| **Gene** | **Species** | **Forward primer** | **Reverse primer** |
| --- | --- | --- | --- |
| *Ins* | Mouse | 5’-CCGCTACAATCAAAAACCATC-3’ | 5’- CAAAGGTGCTGCTTGACAAA-3’ |
| *Gcg* | Mouse | 5’- ACCAGCGACTACAGCAAATA-3’ | 5’-CATGCCTCTCAAATTCATCA-3’ |
| *Ccna2* | Mouse | 5’-AGTCCTTGCTTTTGACTTGG-3’ | 5’- TACGGGTCAGCATCTATCAA-3’ |
| *Ccnb1* | Mouse | 5’-TGCTCTTGGAGACATTGGTA-3’ | 5’-CAGGTTTTGGTAGGGCTTTA-3’ |
| *Ccnb2* | Mouse | 5’-TGCAAGATCGAGGACATAGA-3’ | 5’- GAAACTTGGAATGGACTTGG-3’ |
| *Ccnd1* | Mouse | 5’-AAGATGAAGGAGACCATTCC-3’ | 5’-TTGAGCTTGTTCACCAGAAG-3’ |
| *Ccnd2* | Mouse | 5’-CTATTTCAAGTGCGTGCAGA-3’ | 5’-CAAGAAACGGTCCAGGTAAT-3’ |
| *Il6* | Mouse | 5’-GAGGATACCACTCCCAACAGACC-3’ | 5’-AAGTGCATCATCGTTGTTCATACA-3’ |
| *Tnfa* | Mouse | 5’-CATCTTCTCAAAATTCGAGTGACAA-3’ | 5’- TGGGAGTAGACAAGGTACAACCC-3’ |
| *Ifng* | Mouse | 5’-GCGTCATTGAATCACACCTG-3’ | 5’-GACCTGTGGGTTGTTGACCT-3’ |
| *Il1b* | Mouse | 5’-GTTGATTCAAGGGGACATTA-3’ | 5’- AGCTTCAATGAAAGACCTCA-3’ |
| *Il10* | Mouse | 5’-AGAAATCAAGGAGCATTTGA-3’ | 5’-ATTCATGGCCTTGTAGACAC-3’ |
| *Tnfrsf11b* | Mouse | 5’-TGAGTGTTTTGGTGGACAGT-3’ | 5’-TGCTTTCACAGAGGTCAATG-3’ |
| *CypA* | Mouse | 5’GGCGGCAGGTCCATCTACG-3’ | 5’-CTTGCCATCCAGCCATTCAGTC-3’ |
